# Why Learning How to Chase Butterflies Matters: A Response to Recent Commentaries

**DOI:** 10.15171/ijhpm.2017.114

**Published:** 2017-09-19

**Authors:** Pascale Lehoux, Fiona A. Miller, Geneviève Daudelin, Jean-Louis Denis

**Affiliations:** ^1^Department of Health Management, Evaluation and Policy, School of Public Health, University of Montreal, Montréal, QC, Canada.; ^2^Institute of Public Health Research of University of Montreal (IRSPUM), Montréal, QC, Canada.; ^3^Institute of Health Policy, Management and Evaluation, University of Toronto, Toronto, ON, Canada.

My colleagues and I were honoured to have so many reputable scholars taking precious time to read our article^1^ and write three solid commentaries.^2-4^ We were flattered by their positive remarks and respectfully pondered the weaknesses and omissions their commentaries underlined. In this response, we wish to provide some background to the program of research our article synthesised and reflect on ways to consolidate further research on the value of innovation, which indeed remains a “bright elusive butterfly.”^2^

When we developed a decade ago our research program, we had a hard time to convince our peers that such research was feasible and that it would, ultimately, bring a relevant contribution to health services and policy research. No matter the topic, it is never easy to obtain peer-reviewed funding. Yet, the additional difficulties we faced later when it was time to publish our findings revealed how tricky it is, in practice, to cross disciplinary boundaries and address the “big picture” of innovation in health, which entails understanding how the “supply” works.

On the one hand, when we submitted our findings to journals whose audience were entrepreneurship and innovation policy scholars, we were told that the “health” implications we were seeking to tackle were not important: what really mattered amounted to the entrepreneurial dynamics that supported or hindered the production of innovation. On the other hand, when we submitted our findings to journals whose audiences were health services and policy scholars, we were told that the business and financial dynamics we were describing were alien to health policy: what really mattered was examining the impact of innovation utilisation.

When we received constructive criticisms (and also praise) from six reviewers on what we considered the last piece of our research program we were thus at a loss. A quizzical yet gratifying kind of loss. What happened in the past decade that now makes our research relevant and timely in the eyes of our peers?

One observation by Greenhalgh et al^2^ sheds some light on this question. Throughout their fieldwork on electronic patient records and assisted living technologies that spanned several years, they noticed an emerging “radical change in the strategy taken by large technology companies” to developing new health technologies. Companies such as Microsoft, Tunstall and Philips would show an increasing willingness to:

*engage in long-term strategic partnerships with health and social care organisations, promote open standards, data exchange and interoperability in ways that facilitate collaboration across suppliers and increase potential for widespread adoption, undertake ethnographic studies and co-design projects, and hire clinical staff with extensive patient-facing experience*.

The information technology (IT) sector does indeed open up new ways of designing and bringing new technology to market. It also calls for significant changes in established regulatory processes and reimbursement systems. While the broader medical device industry will not be swiftly transformed by the new IT-based ways of doing business, there is a different breed of entrepreneurs with whom health services and policy researchers could productively collaborate. Similarly, Buttigieg and van Hoof^3^ stress that the design of innovation should mobilise to a greater extent the professional values of clinician-entrepreneurs. Clinicians who anticipate usability, ethical, social and cultural issues and enterprises that show a genuine interest in addressing system-level challenges do in fact contribute to increase the relevance and timeliness of research on the production of innovation.^5^

The model created by Greenhalgh and colleagues that expands our own framework to embrace additional sources of influence forms an excellent basis to pursue research on the value of innovation. Within this perspective, one methodological lesson that we would like to share is not to overstate the difficulty of doing research with entrepreneurs and investors. It is of course challenging because ventures are small enterprises that struggle with steady growth, multiple pressures and commercial secrecy issues. Nevertheless, the entrepreneurs and investors we interviewed were surprised and thrilled that “health folks” showed interest in their work and ways of thinking. They were eager to share with us their knowledge and genuinely voiced their impressions about what required improvements in the innovation ecosystem. In return, our analyses took their business concerns seriously; that is, we carefully sought to identify the rules of the game that condition how they think, what they value and how they handle power relations.

As our article showed, the “butterfly” that clinician-entrepreneurs chase entails a double promise wherein the innovation must come with a profitable business model and generate health benefits. The “butterfly” investors chase is speculative; how much and how fast can a new venture’s economic value grow and generate returns? This is in contrast with the “butterfly” chased by regulators for whom value requires evidence of safety and efficacy (in the case of high-risk devices) and of the legal auditability of its manufacturer. While it is not part of the regulatory agencies’ mandate to consider the relevance of an innovation and its economic implications, these agencies provide economic worth to the ventures that obtain market approval. Hence, by learning how to chase butterflies, we were able to show that those who invest in ventures extract economic value from the regulatory process, an important lesson considering that regulatory requirements are typically characterized as hindering the innovation process.

As Parvizi and Parvizi^4^ underscore, policy-oriented initiatives that build on health technology assessment (HTA) may increase, at an early stage, the circulation of information between technology developers and health decision-makers. For instance, the National Institute for Health and Care Excellence (NICE) in the United Kingdom “have a dedicated process for selecting technologies identified” by the National Institute of Health Research Horizon Scanning Centre and “are informed of new drugs in development 20 months ahead of marketing authorisation.”^5^ Yet, these authors also stress that a more comprehensive health innovation policy is needed, one that entails the “development and maintenance of a resilient and adaptable infrastructure so that the healthcare system is not overwhelmed by new technologies” and on innovation performance measures to “guide future advances.” We could not agree more and invite both health and innovation policymakers to add to their must-read list the review of Nightingale and Coad on the political and methodological biases that plague entrepreneurship research.^6^

## Ethical issues

Not applicable.

## Competing interests

Authors declare that they have no competing interests.

## Authors’ contributions

All co-authors have critically revised this correspondence and approved the final version.

## Authors’ affiliations

^1^Department of Health Management, Evaluation and Policy, School of Public Health, University of Montreal, Montréal, QC, Canada. ^2^Institute of Public Health Research of University of Montreal (IRSPUM), Montréal, QC, Canada. ^3^Institute of Health Policy, Management and Evaluation, University of Toronto, Toronto, ON, Canada.

## References

[1] Lehoux P, Miller F, Daudelin G, Denis J (2017). Providing value to new health technology: the early contribution of entrepreneurs, investors, and regulatory agencies. Int J Health Policy Manag.

[2] Greenhalgh T, Fahy N, Shaw S. The bright elusive butterfly of value in health technology development: Comment on “Providing value to new health technology: the early contribution of entrepreneurs, investors, and regulatory agencies.” Int J Health Policy Manag. 2017; forthcoming. 10.15171/ijhpm.2017.65 PMC5745872 29325407

[3] Buttigieg S, Van Hoof J. The conceptualization of value in the value proposition of new health technologies; Comment on “Providing value to new health technology: the early contribution of entrepreneurs, investors, and regulatory agencies.” Int J Health Policy Manag. 2017; forthcoming. 10.15171/ijhpm.2017.75 PMC5819379 29524943

[4] Parvizi N, Parvizi S. New Health Technologies: A UK Perspective; Comment on “Providing Value to New Health Technology: The Early Contribution of Entrepreneurs, Investors, and Regulatory Agencies.” Int J Health Policy Manag. 2017; forthcoming. 10.15171/ijhpm.2017.59 PMC5726322 29172379

[5] Designing Responsible Innovation in Health: The In Fieri Research Program. http://infieri.umontreal.ca/en/home/.

[6] Nightingale P, Coad A (2014). Muppets and Gazelles: Political and Methodological Biases in Entrepreneurship Research. Ind Corp Change.

